# A New Risk Variant for Multiple Sclerosis at 11q23.3 *Locus* Is Associated with Expansion of *CXCR5+* Circulating Regulatory T Cells

**DOI:** 10.3390/jcm9030625

**Published:** 2020-02-26

**Authors:** Elia Gil-Varea, Maria Fedetz, Herena Eixarch, Nino Spataro, Luisa María Villar, Elena Urcelay, Albert Saiz, Óscar Fernández, Laura Leyva, Lluís Ramió-Torrentà, Koen Vandenbroeck, David Otaegui, Tamara Castillo-Triviño, Guillermo Izquierdo, Sunny Malhotra, Elena Bosch, Arcadi Navarro, Antonio Alcina, Xavier Montalban, Fuencisla Matesanz, Manuel Comabella

**Affiliations:** 1 Servei de Neurologia-Neuroimmunologia, Centre d’Esclerosi Múltiple de Catalunya (Cemcat), Institut de Recerca Vall d’Hebron (VHIR), Hospital Universitari Vall d’Hebron, Universitat Autònoma de Barcelona, 08035 Barcelona, Spain; egilva.90@gmail.com (E.G.-V.); herena.eixarch@vhir.org (H.E.); sunnymalhotra4u24@gmail.com (S.M.); xavier.montalban@cem-cat.org (X.M.); 2 Department of Cell Biology and Immunology, Instituto de Parasitología y Biomedicina “López Neyra”, Consejo Superior de Investigaciones Científicas (IPBLN-CSIC), 18016 Granada, Spain; mfedetz@gmail.com (M.F.); pulgoso@ipb.csic.es (A.A.); 3 Genetics Laboratory, UDIAT-Centre Diagnòstic, Parc Taulí Hospital Universitari, Institut d’Investigació i Innovació Parc Taulí I3PT, Universitat Autònoma de Barcelona, 08208 Sabadell, Spain; nspataro@tauli.cat; 4 Departments of Immunology and Neurology, Multiple Sclerosis Unit, Hospital Ramon y Cajal, (IRYCIS), 28034 Madrid, Spain; luisamaria.villar@salud.madrid.org; 5 Lab. of Genetics of Complex Diseases, Hospital Clinico San Carlos, Instituto de Investigacion Sanitaria San Carlos (IdISSC), 28040 Madrid, Spain; elena.urcelay@salud.madrid.org; 6 Servicio de Neurología, Hospital Clinic and Institut d’Investigació Biomèdica Pi i Sunyer (IDIBAPS), Universitat de Barcelona, 08036 Barcelona, Spain; ASAIZ@clinic.cat; 7 Unidad de Gestión Clínica de Neurociencias, Instituto de Investigación Biomédica de Málaga (IBIMA), Hospital Regional Universitario de Málaga, Universidad de Málaga, 29010 Málaga, Spain; oscar.fernandez.sspa@gmail.com (Ó.F.); laura.leyva.exts@juntadeandalucia.es (L.L.); 8 Girona Neuroimmunology and Multiple Sclerosis Unit, Neurology Department, Dr. Josep Trueta University Hospital, Neurodegeneration and Neuroinflammation Group, Girona Biomedical Research Institute (IdIBGi), Department of Medical Sciences, Faculty of Medicine, University of Girona, 17190 Girona, Spain; llramio@idibgi.org; 9 Inflammation & Biomarkers Group, Biocruces Bizkaia Health Research Institute, 48903 Barakaldo, Spain; k.vandenbroeck@ikerbasque.org; 10 IKERBASQUE, Basque Foundation for Science, 48013 Bilbao, Spain; 11 Neurosciences Area, Biodonostia Health Research Institute, 20014 San Sebastián, Spain; David.otaegui@biodonostia.org; 12 Servicio de Neurología, Hospital Universitario Donostia, 20014 San Sebastián, Spain; tamaracastillo.tri@gmail.com; 13 Departamento de Neurología, Hospital Universitario Virgen Macarena, 41009 Sevilla, Spain; g.i.ayuso@gmail.com; 14 Institute of Evolutionary Biology (CSIC-UPF), Department of Experimental and Health Sciences, Universitat Pompeu Fabra, 08003 Barcelona, Spain; elena.bosch@upf.edu (E.B.); arcadi.navarro@upf.edu (A.N.); 15 Centro de Investigación Biomédica en Red de Salud Mental (CIBERSAM), 43200 Reus, Spain; 16 Centre de Regulació Genòmica (CRG), 08003 Barcelona, Spain; 17 Institució Catalana de Recerca i Estudis Avançats (ICREA), 08010 Barcelona, Spain; 18 Center for Multiple Sclerosis, St. Michael’s Hospital, University of Toronto, Toronto, ON M5S 1A1, Canada

**Keywords:** multiple sclerosis, genetics, targeted DNA sequencing, genotyping, single nucleotide polymorphisms, *CXCR5*

## Abstract

Genome-wide association studies and meta-analysis have contributed to the identification of more than 200 *loci* associated with multiple sclerosis (MS). However, a proportion of MS heritability remains unknown. We aimed to uncover new genetic variants associated with MS and determine their functional effects. For this, we resequenced the exons and regulatory sequences of 14 MS risk genes in a cohort of MS patients and healthy individuals (*n* = 1070) and attempted to validate a selection of signals through genotyping in an independent cohort (*n* = 5138). We identified three new MS-associated variants at C-X-C motif chemokine receptor 5 (*CXCR5*), Ts translation elongation factor, mitochondrial (*TSFM*) and cytochrome P450 family 24 subfamily A member 1 (*CYP24A1*). Rs10892307 resulted in a new signal at the *CXCR5* region that explains one of the associations with MS within the *locus*. This polymorphism and three others in high linkage disequilibrium mapped within regulatory regions. Of them, rs11602393 showed allele-dependent enhancer activity in the forward orientation as determined by luciferase reporter assays. Immunophenotyping using peripheral blood mononuclear cells from MS patients associated the minor allele of rs10892307 with increased percentage of regulatory T cells expressing CXCR5. This work reports a new signal for the *CXCR5* MS risk *locus* and points to rs11602393 as the causal variant. The expansion of CXCR5+ circulating regulatory T cells induced by this variant could cause its MS association.

## 1. Introduction

Increasing evidence indicates that both genetic and environmental factors, such as cigarette smoking, vitamin D insufficiency and Epstein-Barr virus, contribute to multiple sclerosis (MS [MIM: 126,200]) susceptibility [1]. Numerous studies suggest that gene-environment interactions are fundamental to determine the MS patient phenotype [2]. Throughout the last decades, a number of genome-wide association studies (GWAS) and meta-analysis have significantly unmasked more than 200 MS-associated genetic variants outside the major histocompatibility complex at a genome-wide significance level (*p* < 5 × 10^−8^) [3], but a notable percentage of MS heritability remains to be explained. The majority of immune-mediated, disease-associated variants identified are not located within protein-coding regions, but in regulatory sequences, such as gene promoters or enhancers [4]. However, genomic distal elements can interact with their target gene promoters by means of chromatin looping to regulate transcriptional activity [5], which justifies the possible relevance of such non-coding single nucleotide polymorphisms (SNPs) in disease pathogenesis. Furthermore, each identified genetic variant does not necessarily constitute the causal polymorphism responsible for an association signal at a *locus* in GWAS, as it may merely be significant due to linkage disequilibrium (LD) with a causal one. In fact, in MS most of the causal variants, as well as their underlying mechanisms of action, remain largely unknown [6] and specific studies are required to elucidate their potential functional role. What is known is that every major immune cell type is enriched for MS susceptibility genes and that MS risk variants are enriched in some types of brain immune cells [3]. Specifically, there is GWAS evidence supporting the relevance of T follicular helper cells (Tfh) in MS by identifying polymorphisms in the Tfh genes interleukin 21 (*IL-21*), C-X-C motif chemokine receptor 5 (*CXCR5*), and programmed cell death 1 (*PD-1*) associated with diagnosis or disease prognosis. These cells, which are involved in the formation of germinal centers and the activation, expansion and differentiation of B cells into antibody-producing cells, have been found infiltrated in the central nervous system (CNS) of MS patients, potentially contributing to the inflammatory activity of pathogenic B-cells and thus constituting a promising target for MS therapies [7]. In addition, regulatory follicular T helper cells (Tfr), a subset of forkhead box P3 (Foxp3) + T regulatory cells which suppress germinal center responses [8] in order to inhibit B cell proliferation and antibody production [9], have also been shown to be deficient in MS patients [7]. In the present study, we first aimed to detect novel SNP-based MS associations within 14 selected MS risk genes by means of DNA resequencing and genotyping and then to identify the causal variant for the strongest association throughout specific functional studies.

## 2. Materials and Methods

### 2.1. MS Patients and Healthy Donors

The DNA resequencing cohort comprised a total of 1070 individuals after applying the sample quality control exclusion criteria, of which 524 were patients with MS and 546 were age- and sex-matched healthy donors (HD) of European origin. All individuals included in the DNA resequencing cohort were recruited from 5 Spanish MS centers. Supplementary Table S1 summarizes demographic characteristics of the DNA resequencing cohort. The validation cohort consisted of 3450 MS patients and 1688 age- and sex-matched HD of European origin who passed the genotyping quality control, all of them recruited from 8 Spanish MS centers. Supplementary Table S2 abridges demographic characteristics of the validation cohort.

### 2.2. Targeted DNA Resequencing

A total of 14 candidate genes previously associated with MS susceptibility by different GWAS [3,10,11] were selected based on the fulfillment of one or two of the following criteria: (i) their differential expression between MS cases and HD observed in gene expression microarray studies performed by our group using peripheral blood mononuclear cells (PBMC) (unpublished data; criterion 1; see Supplementary Methods); (ii) their potential role in MS pathogenesis according to the literature (criterion 2). The 14 genes selected for DNA resequencing were Fc receptor like 1 (*FCRL1* [MIM: 606,508]; criterion 2: [12]), regulator of G protein signaling 1 (*RGS1* [MIM: 600,323]; criterion 1: up-regulated in MS patients versus HD; criterion 2: [13]), translocase of inner mitochondrial membrane domain containing 1 (*TIMMDC1* [MIM: 615,534]; criterion 1: down-regulated in MS patients versus HD), hematopoietically expressed homeobox (*HHEX* [MIM: 604,420]; criterion 1: down-regulated in MS patients versus HD), *CXCR5* ([MIM: 601,613]; criterion 2: [14]), lymphotoxin beta receptor (*LTBR* [MIM: 600,979]; criterion 2: [15]), Ts translation elongation factor, mitochondrial (*TSFM* [MIM: 604,723]; criterion 2: [16]), galactosylceramidase (*GALC* [MIM: 606,890]; criterion 2: [17]), TNF receptor associated factor 3 (*TRAF3* [MIM: 601,896]; criterion 1: up-regulated in MS patients versus HD), signal transducer and activator of transcription 3 (*STAT3* [MIM: 102,582]; criterion 2: [18]), TNF superfamily member 14 (*TNFSF14* [MIM: 604,520]; criterion 1: down-regulated in MS patients versus HD; criterion 2: [19]), IFI30 lysosomal thiol reductase (*IFI30* [MIM: 604,664]; criterion 2: [20]), CD40 molecule (*CD40* [MIM: 109,535]; criterion 2: [21]) and cytochrome P450 family 24 subfamily A member 1 (*CYP24A1* [MIM: 126,065]; criterion 2: [22]).

A Nimblegen custom array was designed to capture all coding and regulatory regions for each targeted gene. For that, coordinates for each possible exon were obtained from BioMart (Ensembl genes 67, GRCh37.p7) and then extended in order to include putative splicing sites (with 100 intronic base pairs at both ends of each exon) and any possible regulatory sequence (retrieved from Ensembl regulatory build GRCh37) overlapping them. In addition, we also targeted 2000 bp upstream from the transcription start site of each gene to capture the promoter region and end up submitting a total of 140,144 target bases to design. Genomic DNA from peripheral blood samples of the 524 MS patients and the 546 HD from the DNA resequencing cohort was obtained using standard methods and used to construct the Illumina HiSeq DNA libraries following manufacturer’s recommendations for subsequent enrichment with a Nimblegen array. After enrichment, all captured DNA fragments (~140 kb) were sequenced in the DNA resequencing cohort with the Illumina Hiseq technology at BGI Hong Kong (now BGI Tech Solutions). Raw reads were first mapped to the human reference genome (hg19) using the BWA aligner and subsequently processed using the GATK pipeline. Variant discovery and functional annotation were performed with the Haplotype Caller of GATK and ANNOVAR, respectively. Population substructure within MS cases and HD was investigated by means of principal component analysis (PCA). PCA was performed using SmartPCA software package by outputting the first 10 principal components (–k 10). Only 1 sample was found as an outlier over the first component and was removed from further analysis, while 4 samples were found as outliers over the 5th component and were also removed from further analysis. To test the presence of related individuals, we counted the number of genotype differences at all the called variants between each possible pair of samples. A total of 42 sample pairs were showing an extremely low number of differences and for these pairs we discarded the sample with the lowest coverage. Common variants, with minor allele frequencies (MAF) >1%, were tested for association using Fisher’s exact test under an additive model and by calculating asymptotic *p*-values.

### 2.3. Selection of Candidate SNPs for Validation

For prioritization and selection of the top variants, the following steps were taken: (i) discard of variants associated with MS in previous studies; (ii) analysis of LD between our significant SNPs and selection of a representative tag SNP when appropriate based on p-value and odds ratio (OR); (iii) exclusion of SNPs with LD *r*^2^ > 0.3 between the tag SNPs and those in the same region previously reported as associated with MS in the last published meta-analysis [3]; (iv) assessment of SNPs compatibility for the multiplex PCR reaction included in the genotyping process; (v) inclusion of variants with relevant functional potential. A total of 10 variants located at 8 different MS susceptibility genes passed the selection process and thus were selected for validation by means of genotyping. Furthermore, 8 additional variants selected from the last International MS Genetics Consortium (IMSGC) meta-analysis [3] and located in the same genomic region as ours were also included to perform conditional logistic regression analyses if required.

### 2.4. SNPs Genotyping

In a second or validation phase of the study, the 10 selected SNPs from the DNA resequencing phase were genotyped in an independent cohort composed of 3450 patients with MS and 1688 HD. Genomic DNA from peripheral blood samples of included individuals was obtained using standard methods. SNP genotyping was performed by the Agena Bioscience MassARRAY platform (San Diego, CA, USA) and the iPLEX^®^ Gold chemistry at The Spanish National Genotyping Center (CeGen-PRB2, USC node). Genotyping assays were designed using the Agena Bioscience MassARRAY Assay Designer 4.1 software (San Diego, CA, USA). Briefly, targeted amplification of fragments containing the SNPs (~100 bp) by means of multiplex PCR was followed by allelic discrimination via single-base extension (SBE) reactions to generate products of different molecular mass detectable by MALDI-TOF mass spectrometry. A detector was coupled to the MassARRAY Typer 4.0 software (San Diego, CA, USA), which calculated the products mass and thus determined the base. The assay was performed in a 384-well plate which included negative controls and a trio of Coriell samples (Na10860, Na10861 and Na11984) for quality control. Random samples (5%) were tested in duplicate and the reproducibility was 100%. Genotyping data was analyzed using PLINK V.1.05. In order to determine the effect and independence of variants association with MS, logistic regression models were computed. The validity of our control cohort was checked by Hardy–Weinberg equilibrium (HWE). Disease-free control groups from outbred populations should follow the HWE. Departure from Hardy-Weinberg equilibrium for all SNPs was tested using an exact test [23]. LD patterns between SNPs were analyzed with Haploview 4.2 (Broad Institute, Cambridge, MA, USA) [24] using a European population from 1000 Genomes.

### 2.5. Luciferase Reporter Assays

Eight fragments in both forward and reverse orientations with potential enhancer activity, as indicated by ENCODE, and containing either the minor or the reference allele of the four variants under study (rs11602393, rs55756957, rs10892307 and rs3176905, 16 inserts in total) were subjected to a dual-luciferase reporter assay. For that purpose, each fragment was PCR amplified by means of specific primers design (see Supplementary Table S3 for oligonucleotides sequences, coordinates hg19 of each insert, and fragment size) using DNA from a heterozygote individual for all variants, aiming to obtain both the alternative and the wild-type amplicons for each SNP. Fragments were independently cloned into the pCR™8/GW/TOPO vector backbone and sequenced. Then, each clone was inserted in forward and reverse orientation in the pGL4.23-GW [*luc2*/minP] vector, containing a minimal promoter upstream of the luciferase reporter gene. pGL4.23-GW [*luc2*/minP] contains a TATA-box promoter element immediately upstream of the luciferase reporter gene and immediately downstream of the multiple cloning region. The minimal TATA promoter has low basal activity and allows for sensitive response element activity measurements. HEK-293 cells were transfected in quadruplicate for each construct. The HEK-293 cell line was maintained in DMEM culture medium supplemented with 2.0 mM L-Glutamine, 10% decomplemented Fetal Bovine Serum (FBS) and 1% non-essential amino acids (Gibco BRL, Life Technologies LTD, Paisley, UK). Cells were transfected using the jetPRIME^®^ polymer-based reagent (Polyplus-Transfection, NY, USA) according to the manufacturer’s instructions. Briefly, 60%–80% confluent HEK-293 cultures were mixed either with 0.1 µg of a given construct or with 0.1 µg of pGL4.23-GW empty plasmid as negative control and 0.2 ng pRL-TK renilla *luc* internal control (Promega) for normalizing transfection efficiency. After 24 h of incubation in 96-well plates at 37 °C, cells were washed twice in Phosphate Buffered Saline (PBS) at 4 °C and firefly and renilla luciferase activities were evaluated in 40 µg of protein from cell supernatants using Dual Luciferase^®^ Reporter Assay System kit (Promega) and a luminometer F12 (Berthold Detection Systems). Results both from each construct and from the empty vector were normalized by dividing the luciferase reporter activity by the renilla reporter activity. Fold changes were calculated by dividing each normalized construct by the normalized empty vector. Quadruplicate samples were then averaged.

### 2.6. Determination of Gene Expression Levels in PBMC

Messenger RNA expression levels for *CXCR5* were determined in PBMC from a subgroup of untreated MS patients recruited from the genotyping cohort classified according to the presence (*n* = 15) or absence (*n* = 18) of the minor allele for rs10892307 (Supplementary Table S4 summarizes demographic and clinical information). PBMC from MS patients were isolated by Ficoll-Isopaque density gradient centrifugation (Gibco BRL, Life Technologies LTD, Paisley, UK) and stored in liquid nitrogen until used. Total RNA was extracted from PBMC using TRIzol (GIBCO-BRL, Life Technologies, Grand Island, NY, USA) and cDNA synthesized using the High-Capacity cDNA Reverse Transcription Kit (Applied Biosystems, Foster City, CA, USA). *CXCR5* expression levels were determined by real-time PCR using a specific TaqMan^®^ probe (Hs00540548_s1, Applied Biosystems). The housekeeping gene glyceraldehyde-3-phosphate dehydrogenase (*GAPDH*) was used as endogenous control (Applied Biosystems). Assays were run on the ABI PRISM® 7900HT system (Applied Biosystems) and data were analyzed with the 2^−ΔΔCT^ method [25]. Results were expressed as fold-change in gene expression in rs10892307 minor allele carriers to those non-carriers and represented in graphs as delta threshold cycle values, which are inversely related to quantity.

### 2.7. Flow Cytometry

A total of 16 untreated patients with MS classified according to the presence (*n* = 8) or absence (*n* = 8) of the rs10892307 SNP minor allele (G) were selected either from the DNA resequencing cohort or from the genotyping cohort. PBMC from MS patients were obtained and stored as described above (demographic and clinical information is summarized in Supplementary Table S5). A total of 1 × 10^6^ cells were stained with different antibody cocktails at 4 °C for 30 min to assess the cell surface expression of CXCR5 in different circulating T and B lymphocytes subpopulations by means of flow cytometry. Cells were surface stained using the following antibodies (from BD Biosciences, San Diego, CA, USA) unless indicated otherwise): anti-human CD3-FITC, anti-human CD4-APC·H7, anti-human CD45RO-BV605, anti-human CD25-BV421 (Biolegend, San Diego, CA, USA), anti-human CD19-PerCP·Cy5.5, anti-human CD20-APC·H7, anti-human CD27-BV605, anti-human CD38-PE, anti-human CD24-BB515 and anti-human CXCR5-Alexa Fluor^®^ 647. Intranuclear staining for Foxp3 was performed after surface staining using a commercial kit (eBiosciences Vienna, Austria). Briefly, cells were washed, permeabilized and fixed according to the manufacturer’s instructions and then stained with anti-human Foxp3-PE (eBiosciences) at room temperature for 30 min. Cells were washed, resuspended in PBS (with 1% BSA and 0.1% sodium azide) and acquired on a CytoFLEX flow cytometer (Beckman Coulter, Brea, CA, USA). The CytExpert 1.0 software (Beckman Coulter, Brea, CA, USA) was employed for analysis. Cells were gated based on forward and side scatter properties and T and B cells subpopulations subsequently based on their cell surface markers (Supplementary Figure S1 summarizes the gating strategy). The percentage of CXCR5+ cells was calculated for each subpopulation from each included MS patient. Median fluorescence intensity (MFI) of cells expressing CXCR5 was calculated by subtracting the Alexa Fluor^®^ 647 MFI value of a non-stained control from the Alexa Fluor^®^ 647 MFI value of each sample.

### 2.8. Statistical Analysis

DNA resequencing and SNPs genotyping statistical analysis is explained in their respective sections. A Mann-Whitney U test was employed to test for significant differences in: (i) luciferase activities between different constructs, as well as between constructs and the pGL4.23-GW empty plasmid; (ii) *CXCR5* mRNA expression levels in PBMC between MS patients with and without the minor allele (G) of the rs10892307 variant; and (iii) the proportion of CXCR5+ cells from different peripheral T and B lymphocytes subpopulations between MS patients carriers and non-carriers of the rs10892307 minor allele (G).

### 2.9. Standard Protocol Approvals, Registrations, and Patient Consents

Written informed consent was obtained from all subjects and the design of the work was approved by the local Ethics Committees of the different participating institutions (Comité Ético de Investigación Clínica del Hospital Clínico San Carlos, with code 16/211-E; Comité Ético de Investigación de los Hospitales Universitarios Virgen Macarena y Virgen del Rocío, code 43160035; Comité Ético de Investigación Clínica de Euskadi, with code CEI-CE300911; Clinical Research Ethics Committee of the Vall d’Hebron Hospital, with code EPA(AG)57/2013(3834); Comité Ético de Investigación Clínica del Hospital Clinic, with code HCB/2015/0236). Research was conducted in accordance with the principles of the Declaration of Helsinki.

## 3. Results

### 3.1. Targeted Resequencing of MS Risk Genes in the Discovery Cohort Unmasks 32 SNPs Associated with MS Susceptibility

With the objective of identifying new common genetic variants associated with MS susceptibility, we first performed targeted resequencing of 14 selected MS risk genes in a discovery cohort composed of 524 MS patients and 546 HD (*n* = 1070). The average depth of coverage of both the samples and the resequenced fragments was 85.64×. As shown in Table 1, a total of 32 SNPs within nine susceptibility genes were differentially distributed between MS patients and HD (exact *p*-values < 0.05). Three of these SNPs have been previously reported as MS associated variants (rs4796791, rs4810485, rs2248137) [3,13] (Table 1). Of these, the minor alleles of 13 polymorphisms were found to be more frequently represented in patients with MS than in HD (OR > 1.0), which classifies them as variants associated with an increased risk for MS, while 19 were more frequently detected in HD than in MS cases (OR < 1.0) which supports their protective role against MS. Based on the selection criteria detailed in the Methods section, 10 polymorphisms in eight loci were chosen from the discovery cohort for validation in an independent cohort of MS patients and HD. In order to test whether the reported SNPs constituted newly identified MS-associated signals, eight additional polymorphisms associated with MS and located within the same genomic region of our selected variants were also incorporated in the genotyping phase for the purpose of determining whether they were independent signals. A description of selected SNPs from the discovery cohort and from previously reported studies are summarized in Table 2.

### 3.2. Validation of Selected SNPs in an Independent Cohort

Selected variants were genotyped in an independent cohort of 3450 MS patients and 1688 age- and sex-matched HD (*n* = 5138). As depicted in Table 3, four polymorphisms selected from the DNA resequencing phase were successfully validated in the second phase of the study. The variants showing significant differences between MS patients and HD were the following: rs10892307 (*p* = 1.37 × 10^−6^, OR = 0.77), located at the 5′ untranslated region (5′UTR) of *CXCR5*, two base pairs upstream from the start codon; rs2762943 (*p* = 0.047, OR = 1.16), located at the 5′ upstream region of the *CYP24A1* gene, 672 base pairs upstream from the start codon; rs1599932 (*p* = 0.001, OR = 0.85), located in the *TSFM* gene within a region previously associated with MS susceptibility [16]; and rs3746821 (*p* = 0.03, OR = 1.15), an expression quantitative trait locus (eQTL) for the *CD40* gene. The remaining six SNPs showed a lack of statistically significant associations with MS risk (*p* > 0.05). On the other hand, we could reproduce the previously described associations of five out of the eight reference variants previously reported by the IMSGC 2019.

### 3.3. Conditional Logistic Regression Analysis Reveals rs10892307 in the CXCR5 Locus as a New Independent Signal

In order to determine if the new validated associations were new signals in the loci we performed conditional logistic regression analyses (Table 4). The results showed that the two genotyped SNPs at the *TSFM* locus were capturing the same association signal. The same was obtained for the *CD40* locus, but in this region we observed that the variant reported by the IMSGC [3] explained better the association than the SNP identified in the present work (rs3746821). We observed a more complex situation with the *CXCR5* locus in which the meta-analysis performed by the IMSGC [3] described three independent MS association signals within the *CXCR5* locus (Supplementary Figure S2). Logistic regression analysis of all SNPs conditioning on the three associated SNPs in our cohort revealed that rs10892307 and the already known rs149114341 produced independent signals for association, since both remained significant when the effect from other variants was adjusted (all *p* < 0.05; Table 4). We also observed that rs10892307 better explained the association compared to rs6589706 and seemed to be the primary signal.

### 3.4. Tag-SNP Search of rs10892307 and Analysis within the Region

**Figure 1:**
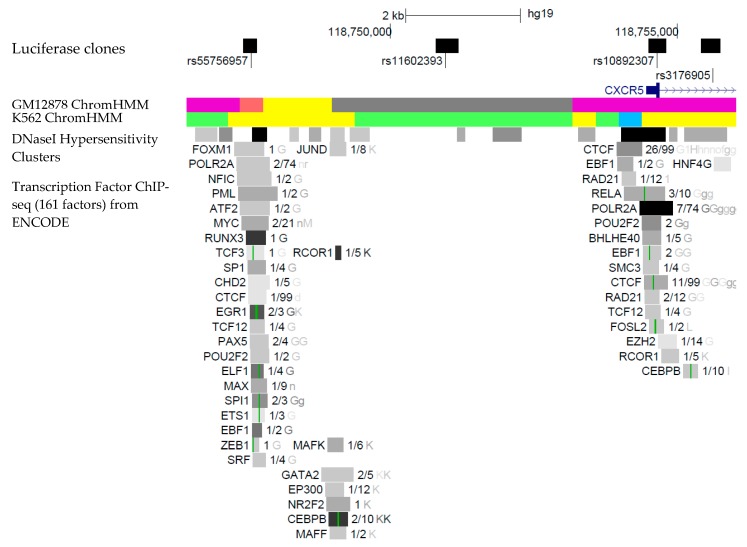
Scheme of the *CXCR5* gene region. The picture shows that variants rs10892307, rs55756957, rs11602393 and rs3176905 are located within potential regulatory regions, as indicated by Encyclopedia of DNA Elements (ENCODE). From top to bottom, the figure represents (i) the scale and the chromosomal position (GRCh37/hg19), (ii) the cloned DNA fragments for the luciferase reporter assay (dark boxes), and (iii) the genomic region where the variants map. (iv) Chromatin State Segmentation by Hidden Markov Model (HMM) from ENCODE/Broad. (v) DNase I Hypersensitivity Clusters track shows regions where chromatin is hypersensitive to the DNase I enzyme. (vi) Transcription Factor ChIP-seq track shows DNA regions where the transcription factors bind to, as assayed by chromatin immunoprecipitation followed by the precipitated DNA sequencing (ChIP-seq). A gray box encloses each peak cluster of transcription factor occupancy, with the darkness of the box being proportional to the maximum signal strength observed in any cell line contributing to the cluster. University of California Santa Cruz (UCSC) Browser was used to construct the scheme (https://genome.ucsc.edu/).

Aiming to find the SNP that may be the causal variant for association of the rs10892307 signal, we identified the tagged SNPs for rs10892307 by analyzing the LD of rs10892307 with the locus variants in the European populations of 1000 Genome Project. We found two SNPs in almost total LD with rs10892307, rs11602393 (*r*^2^ = 0.94; D’ = 0.97), positioned at the 5′ upstream region of the *CXCR5* gene and rs3176905 (*r*^2^ = 0.99; D’ = 1.0), located within the one single intron of *CXCR5.* The next SNP in LD with rs10892307 was rs55756957 (*r*^2^ = 0.54; D’ = 0.94), which was also selected because it was in a regulatory region (Figure 1) predicted by the Encyclopedia of DNA Elements (ENCODE).

To estimate the potential functional effect of each variant we used the annotations of gene regulatory regions from ENCODE. As shown in Figure 1, rs55756957, rs10892307 and rs3176905 were positioned within regions associated with histone modifications, binding sites of different transcription factors and DNase I hypersensitive clusters. Besides, rs55756957 was located in a Weak Promoter and rs10892307 and rs3176905 were located in an Inactive/poised Promoter by the prediction of Hidden Markov Model (HMM) from the ENCODE data for the GM12878 lymphoblastoid cell line. Functional potential of rs11602393 was studied with RegulomeDB [27] browser (http://regulomedb.org/) and found that annotations with ChromHMM with epigenomic information from blood and T-cells predict its location within an enhancer, giving a score of six for regulatory potential to this variant.

### 3.5. Luciferase Reporter Assays Point to rs11602393 as the Casual Variant

**Figure 2:**
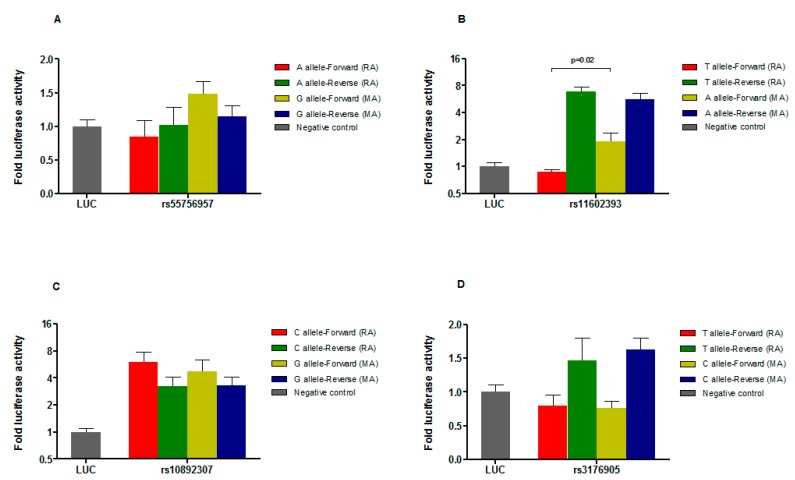
Effect of the variants of the rs10892307 LD block. The graphs show the fold of luciferase activity in relation to the empty vector (negative control) of the different constructs corresponding to the four genomic regions with potential regulatory activity containing the four selected *CXCR5* polymorphisms: (**A**) SNP rs55756957; (**B**) SNP rs11602393; (**C**) SNP rs10892307; and (**D**) SNP rs3176905. For each region, four constructs carrying either the minor (MA) or the reference (RA) allele and either in forward or in reverse orientation are represented. Each bar represents the mean (± standard deviation) of four independent experiments. Luciferase activity levels are referred to those of an internal control vector containing a basic promoter driving expression of the renilla luciferase reporter protein. LUC: empty plasmid. RA: reference allele. MA: minor allele.

In order to experimentally test the potential effect of the selected variants on the regulatory activity of each sequence, we carried out a dual luciferase reporter assay. For each polymorphism we cloned the fragment containing either its major (i.e., reference allele) or its minor allele upstream of the minimal promoter of a firefly luciferase reporter vector both in forward and reverse orientations. Four independent transfection experiments were performed for each reporter vector in human embryonic kidney (HEK-293) cells. As depicted in Figure 2A, the fragment carrying the rs55756957 SNP did not show any enhancer activity in HEK-293 cells. However, constructs carrying the rs11602393, rs10892307 or rs3176905 variants demonstrated enhancer activity. Fragments containing the reference allele either of rs11602393 (Figure 2B) or of rs3176905 (Figure 2D) showed allele-independent enhancer activity only in the reverse orientation. In the forward orientation, we observed significantly increased enhancer activity of the minor allele of rs11602393 when compared with its major allele (*p* = 0.02), showing 2.2-fold higher ratio of luciferase activity. Finally, the fragment containing the variant rs10892307 showed orientation-independent and allele-independent enhancer activity (Figure 2C), although no statistically significant differences were reported between alleles.

### 3.6. rs10892307 Alleles Are Not Associated with Differences in CXCR5 mRNA Expression Levels in PBMC from MS Patients

**Figure 3:**
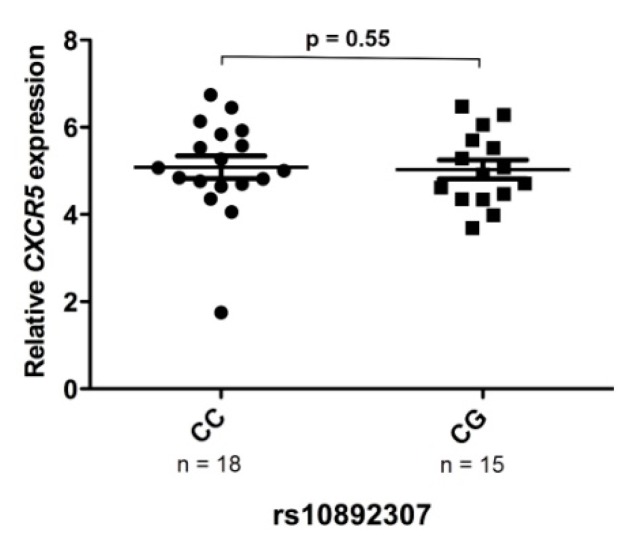
Expression levels of *CXCR5* in MS patients classified according to the presence or absence of the rs10892307 polymorphism minor allele. Graph shows the mRNA expression levels for *CXCR5* determined in peripheral blood mononuclear cells (PBMC) from untreated MS patients by real-time PCR relative quantification. The y-axis represents the delta threshold cycle values, which are inversely related to quantity. The x-axis indicates rs10892307 minor allele carriers (CG; *n* = 15) and non-carriers (CC; *n* = 18).

In order to evaluate whether the minor allele for rs10892307 polymorphism was associated with changes in the *CXCR5* gene expression, mRNA levels of this gene were measured in PBMC from MS patients classified according to the presence or absence of the minor allele for the rs10892307 variant by real-time PCR. As shown in Figure 3, *CXCR5* expression levels were similar between minor allele carriers and non-carriers.

### 3.7. The Percentage of CXCR5 Positive Regulatory T Cells Is Increased in MS Patients Carrying the Minor Allele for rs10892307

**Figure 4:**
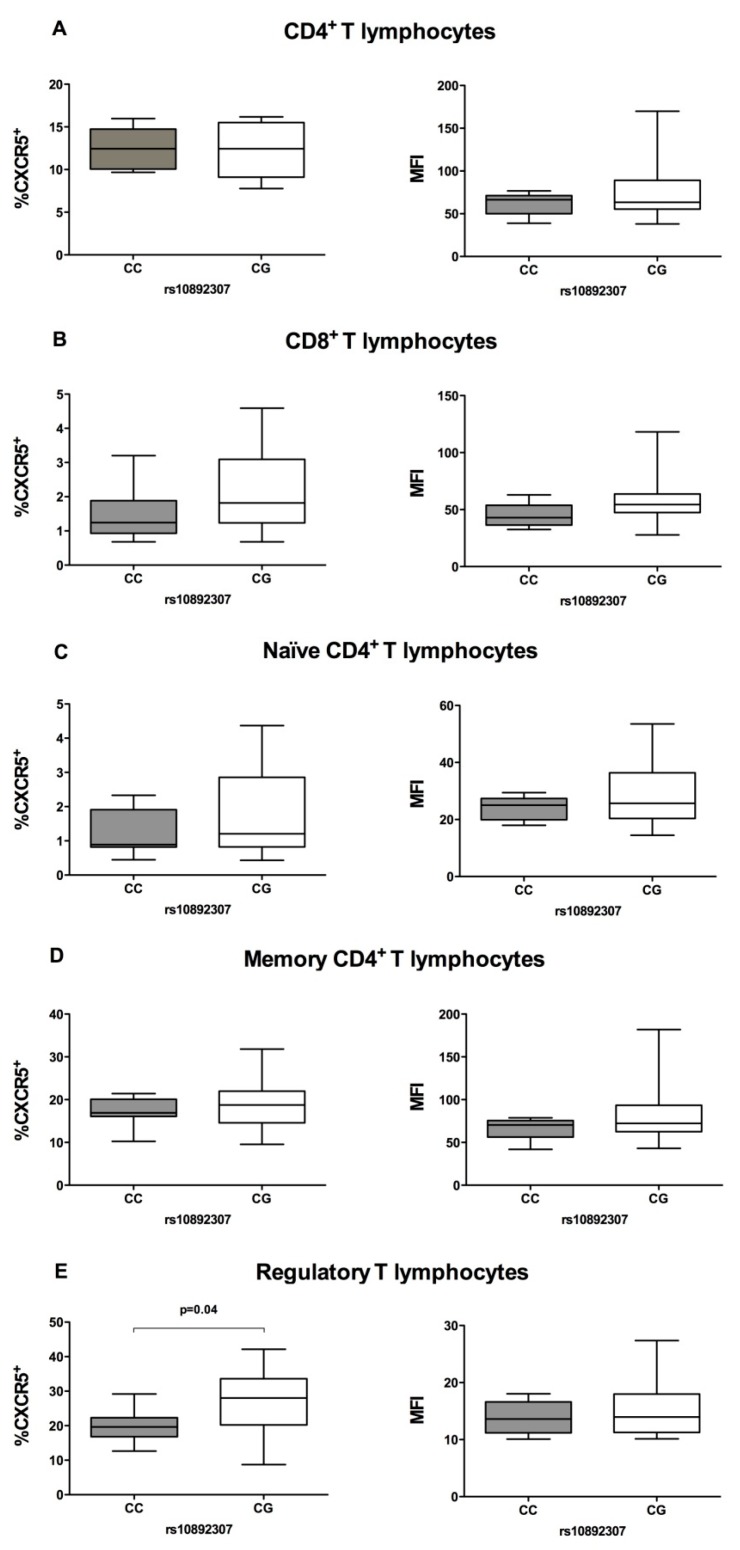
CXCR5 immunophenotyping in peripheral T lymphocytes subpopulations from MS patients classified as rs10892307 minor allele carriers or non-carriers. Graphs show the percentage of CXCR5+ cells (left) and the median fluorescence intensity (MFI) of CXCR5 (right) in (**A**) CD4^+^, (**B**) CD8^+^, (**C**) CD4^+^ naïve, (**D**) CD4^+^ memory and (**E**) regulatory T lymphocytes from MS patients with (CG; *n* = 10) and without (CC; *n* = 10) the rs10892307 minor allele. Boxes represent the 75th and 25th percentiles divided horizontally by the median. Whiskers are drawn to the nearest value not beyond a standard span from the quartiles.

**Figure 5:**
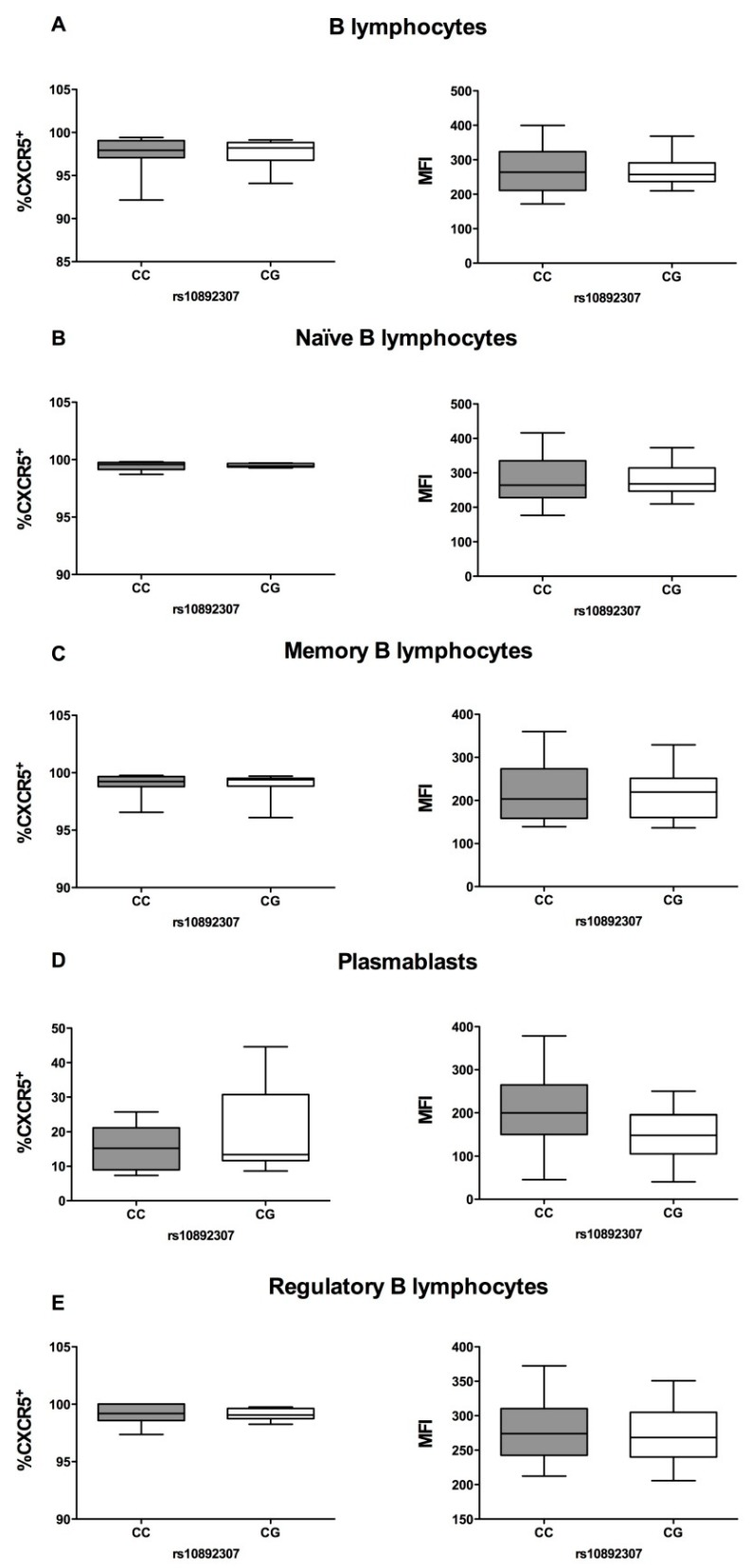
CXCR5 immunophenotyping in peripheral B lymphocytes subpopulations from MS patients stratified according to the presence or absence of the rs10892307 variant minor allele. Graphs depict the percentage of CXCR5+ lymphocytes (left) and the median fluorescence intensity (MFI) of CXCR5 (right) in (**A**) all B cells, (**B**) naïve B cells, (**C**) memory B cells, (**D**) plasmablasts and (**E**) regulatory B lymphocytes from MS patients with (CG; *n* = 10) and without (CC; *n* = 10) the rs10892307 variant minor allele. Boxes represent the 75th and 25th percentiles divided horizontally by the median. Whiskers are drawn to the nearest value not beyond a standard span from the quartiles.

We finally aimed to assess whether the increased promoter transcriptional activity observed for rs11602393 could be translated into differences at the CXCR5 protein level in specific cell populations rather than in the whole set of PBMC. To this end, we used flow cytometry to determine cell surface CXCR5 baseline expression in different populations of T and B lymphocytes from MS patients grouped according to the presence or absence of the minor allele of the rs10892307 polymorphism, given its high LD with the rs11602393 SNP (*r*^2^ = 0.94; D’ = 1.0). We analyzed both the percentage of CXCR5+ cells and the median fluorescence intensity (MFI). As depicted in Figure 4, CXCR5+ circulating regulatory T cells were significantly increased in MS patients carrying the rs10892307 minor allele, that is, the MS-protective allele, compared with minor allele non-carriers (*p* = 0.04). These differences were restricted to the percentage of CXCR5+ cells in regulatory T cells and were not observed for the MFI. Furthermore, CXCR5 expression by CD4^+^ T lymphocytes, naïve CD4^+^ T cells, memory CD4^+^ T cells and CD8^+^ T lymphocytes was similar between both groups of MS patients, both analyzing CXCR5+ cells and MFI. Lastly, as shown in Figure 5, CXCR5 cell surface expression by B cells populations was not affected by the rs10892307 variant.

## 4. Discussion

The *CXCR5* gene was firstly associated with MS susceptibility through the rs630923 polymorphism (C/A) located in its promoter region, whose common allele (C) was associated with an increased risk for MS. This association was established in 2011 (*n* = 27,148), but it did not reach a genome-wide significance *p*-value (*p* = 5 × 10^−8^) [10]. Thus, in 2013, the IMSGC aimed to validate rs630923 association by means of genotyping in an independent cohort of ~20,000 individuals and subsequent meta-analysis of the data from the two studies [28]. Combining the results revealed the association of rs630923 with MS at GWAS significance level. Since that moment, more variants associated with *CXCR5* have been identified as being linked to the risk of developing MS [3,11], findings that further strengthen the relationship of this gene with the disease.

In this study we show a new signal in the *CXCR5* associated *locus*. The associated signals reported to date appeared neither to cause amino acid changes in the proteins encoded in the region, nor to be described as eQTLs in any previous study such as GTEx, Geuvadis or Database of Immune Cell Expression (DICE), which makes it difficult to predict the causal gene of the association and the functional alterations linked to such associated signals. The new signal described here, rs10892307, maps within the 5’ of the *CXCR5* gene, which codes for an outer-membrane chemokine receptor of the CXC family. Under physiological conditions, this receptor is highly expressed in mature B cells and is a key surface antigen to define Tfh populations, both those found in the secondary lymphoid organs and those circulating in the peripheral blood [29]. The CXCR5 unique ligand is the chemokine CXCL13 (also known as BLC, B lymphocyte chemoattractant), which is constitutively expressed in secondary lymphoid organs and attracts CXCR5-positive cells, thus directing homing and motility of such cells [30].

Several studies in the animal model of MS, experimental autoimmune encephalomyelitis (EAE), have proposed an association between CXCL13 and MS. Magliozzi. et al. reported an increased *CXCL13* [MIM: 605,149] gene expression in the CNS, as well as the presence of CXCL13+ cells within abnormal meningeal aggregates organized as B-cell germinal center–like structures [31]. Furthermore, Bagaeva et al. detected CXCL13 mRNA and protein expression in the spinal cord of EAE mice, but not in naïve controls [32]. They also observed that CXCL13^-/-^ MOG-induced EAE mice exhibited a milder disease course, as well as less demyelination compared to wild type mice for CXCL13. Studies linking the CXCL13-CXCR5 axis with MS disease have also been reported in humans. One of them revealed overexpression of the *CXCL13* gene in active demyelinating lesions areas, the presence of the CXCL13 protein both in perivascular and parenchymal inflammatory infiltrates and elevated levels of CXCL13 in the cerebrospinal fluid (CSF) of patients with relapsing-remitting MS [33]. Moreover, most B cells present in the CSF of MS patients were CXCR5+ [34].

The well-documented association of the CXCR5 function with MS heightened our interest in deciphering whether this new reported signal could be altering the *CXCR5* promoter transcriptional activity. Given that our bioinformatics research revealed that rs10892307, as well as the other three SNPs in high LD with it (r^2^ > 0.5), were located in regulatory regions, it was critical to include all the SNPs set in the assay to increase the possibility of detecting the causal variant of this region associated with MS risk. The fragment containing the rs11602393 (T/A) polymorphism increased the promoter transcriptional activity in reverse orientation, but this increase was similar between the fragment carrying the reference allele (T) and that carrying the minor allele (A). However, it is remarkable that in forward orientation the reference allele of this SNP had no effect on the promoter activity, but the minor allele did. The statistically significant difference between the luciferase reporter activity controlled by the reference allele and that controlled by the minor allele of rs11602393 in forward orientation, together with the fact that no effect was observed associated to the remaining studied polymorphisms, led us to hypothesize that rs11602393 is the causal variant underlying the rs10892307 association with MS susceptibility. This theory is buttressed by the fact that the LD between rs10892307 and rs11602393 is almost complete in European populations (*r*^2^ = 0.94, D’ = 0.97).

In this field, other authors have performed studies using luciferase reporter assays to analyze MS-associated polymorphisms [16]. In fact, in one of them, Ryu et al. studied the impact of the rs630923 polymorphism, the first *CXCR5* SNP associated with MS, on the promoter transcriptional activity. However, they did not observe any difference between alleles [35]. Two years later, Mitkin et al. also employed a luciferase assay to analyze the *CXCR5* gene promoter activity influenced by rs630923 and observed that the minor allele (A) created a MEF2C transcription factor binding site that involved a decreased transcriptional activity in Raji cells [36]. Since rs630923 is in low LD with rs10892307 (*r*^2^ = 0.039), the variant that best capture one of the associations in our population for the CXCR5 region, we believe that the functional effect described in Raji cells [36] must be related to other of the MS association signals of the locus.

Based on our dual-luciferase reporter assays, we wanted to delve deeper into the potential effect of the rs11602393 SNP by conducting additional studies using PBMC from MS patients. Patients were selected based on rs10892307 genotypes, which is proxy of rs11602393 (*r*^2^ = 0.93) [37]. Determination of *CXCR5* mRNA expression levels in PBMC from MS patients with presence and absence of the rs10892307 variant minor allele revealed no differences between groups. This outcome could be attributed to a lack of statistical power, since it is improbable to capture an eQTL starting from a small number of PBMC samples from patients in which the expression of CXCR5 could be specifically regulated and differentially expressed among cell types. This hypothesis was reinforced when we examined the expression of cell surface CXCR5 receptor in different circulating T and B cells subpopulations and observed an increased percentage of regulatory T cells positive for CXCR5 in MS patients carrying the minor allele for rs10892307 (G) with respect to non-carriers. This finding would imply that the minor allele of this variant (G), associated with protection to MS, leads to an increased percentage of regulatory T cells positive for CXCR5.

Regulatory T cells expressing CXCR5 were recently defined as Tfr. The presence of CXCR5 on their surface confers them the ability to migrate to germinal centers within secondary lymphoid tissues by chemoattraction towards CXCL13 [38]. There, the germinal centers reaction occurs, where B cells previously activated by dendritic cells through antigen presentation undergo several rounds of somatic hypermutation, clonal expansion, affinity maturation and isotypic switching to finally differentiate into memory B cells or plasma cells [39]. Germinal center reaction is arbitrated by the action of Tfh cells through direct costimulatory signals and cytokine secretion [40], but it also requires additional regulation by Tfr cells to avoid the unwanted autoantibody production and autoimmune disease development, which is accomplished by follicular B cells suppression and Tfh cell cycle inhibition to block their antibody production [41]. As Tfr cells are not only found in lymphoid organs but also in the periphery [42], and comparable in vitro suppressive capacity has been demonstrated in both cellular populations in humans [43], we considered that the analysis of circulating Tfr cells constituted a representative resource to assess alterations in germinal centers processes. In our study, the significantly decreased percentage of circulating CXCR5+ regulatory T cells observed in MS patients with the rs10892307 risk allele (C) would imply a lower proportion of regulatory T cells with migratory potential. This circumstance could unleash a defective inhibition of the germinal centers reaction, with a consequent lack of control over B cells activation and antibody production. In experiments with mice, it has been noted that the absence of Tfr cells supposes an outgrowth of antigen-nonspecific B cell clones in germinal centers [38]. This data is especially interesting in the field of autoimmune diseases, such as MS. In fact, although the number of studies on circulating Tfr cells in autoimmune diseases is limited, some authors suggest that deregulation of Tfr cells could trigger the development of a number of autoimmune conditions. For example, Ma et al. detected a decreased number of circulating Tfr cells in patients with systemic lupus erythematosus [SLE, MIM: 152,700] with respect to HD [44]. Similarly, the results of Zheng et al. showed a reduced number of circulating Tfr cells in primary biliary cholangitis [PBC, MIM: 109,720] patients, an autoimmune disease that causes liver damage, compared to HD [45]. In the field of MS, Dhaeze et al. have recently published that both the frequency and the suppressive ability of circulating Tfr cells is decreased in MS patients when compared with healthy individuals [43]. According to our results, the frequency of these cells would be especially low in those MS patients carrying the rs10892307 variant risk allele (C), which prompts us to speculate that the risk allele is associated with a lower limitation of the germinal centers reaction by Tfr cells and, therefore, with a greater probability that antibodies against own antigens are generated.

In summary, we have identified a new association with MS susceptibility in the *CXCR5* gene. Luciferase reporter assays point to rs11602393 as the causal polymorphism of such association and that its MS risk allele confers decreased promoter activity and specifically reduces the percentage of peripheral blood CXCR5+ regulatory T cells, which suggests a defective germinal center reaction regulation with the ensuing risk of autoimmunity. These hypotheses about the mechanism of action for this polymorphism certainly establish the basis for future studies to verify that carrying the rs11602393 risk allele could impair the germinal centers reaction and increase the production of autoantibodies.

## Figures and Tables

**Table 1:** Variants reported to be differentially distributed between multiple sclerosis (MS) patients and healthy donors (HD) by means of targeted DNA resequencing (*n* = 1070).

Gene	SNP	MAF Cases *	MAF Controls *	Exact *p*-Value	Logistic *p*-Value	Asymptotic *p*-Value	OR	L95	U95	GWAS MS SNP LD (*r*^2^/D’)	Criteria Exclusion (E) or Inclusion (I)
*FCRL1*	rs1016849	0.17	0.21	0.023	0.019	0.021	0.77	0.62	0.96	rs2050568 [11] (0.3/1)	E (iii)
*FCRL1*	rs3811024	0.24	0.2	0.019	0.019	0.018	1.28	1.04	1.57	rs2050568 [11] (0.3444/1)	E (iii)
*FCRL1*	rs3811023	0.25	0.20	0.011	0.011	0.010	1.30	1.06	1.6	rs2050568 [11] (0.33/1)	E (iii)
*FCRL1*	rs34370597	0.25	0.21	0.015	0.016	0.015	1.28	1.05	1.57	rs2050568 [11] (0.33/1)	E (iii)
*CXCR5*	rs10892307	0.13	0.18	0.007	0.007	0.007	0.72	0.57	0.92	*r*^2^ > 0.16 +	I (iii)
*CXCR5*	rs3922	0.40	0.45	0.011	0.012	0.011	0.80	0.67	0.95	*r*^2^ > 0.11 +	I (iii)
*CXCR5*	rs581063	0.45	0.50	0.019	0.019	0.017	0.81	0.69	0.96	*r*^2^ > 0.14 +	E (ii)
*CXCR5*	rs11217083	0.45	0.50	0.027	0.026	0.024	0.82	0.69	0.97	*r*^2^ > 0.14 +	E (ii)
*LTBR*	rs3759323	0.13	0.17	0.030	0.030	0.027	0.76	0.60	0.97	rs2364485 [3] (0.02/0.6)	I (iii)
*LTBR*	rs3759324	0.18	0.21	0.044	0.046	0.040	0.80	0.65	0.99	rs2364485 [3] (0.025/0.59)	E (iii)
*TSFM*	rs11172335	0.22	0.26	0.015	0.014	0.014	0.78	0.64	0.95	rs703842 [26] (1/1)	E (iii)
*TSFM*	rs10747783	0.22	0.26	0.011	0.011	0.011	0.77	0.63	0.94	rs703842 [26] (1/1)	E (iii)
*TSFM*	rs34277814	0.25	0.20	0.004	0.004	0.003	1.35	1.11	1.66	rs703842 [26] (0.14/1)	E (iv)
*TSFM*	rs2014886	0.22	0.26	0.025	0.024	0.024	0.79	0.65	0.97	rs703842 [26] (1/1)	E (iii)
*TSFM*	rs1599932	0.22	0.26	0.022	0.020	0.020	0.79	0.65	0.96	rs703842 [26] (1/1)	I (v)
*TSFM*	rs10783847	0.22	0.26	0.018	0.016	0.016	0.78	0.64	0.96	rs703842 [26] (1/1)	E (iii)
*TSFM*	rs10783848	0.22	0.26	0.018	0.016	0.016	0.78	0.64	0.96	rs703842 [26] (1/1)	E (iii)
*TRAF3*	rs74082969	0.016	0.03	0.044	Na	0.042	0.55	0.30	0.99	rs12588969 [3] (0.03/0.77)	I (iii)
*STAT3*	rs141732716	0.02	0.009	0.014	0.013	0.011	2.53	1.20	5.32	rs1026916 [3] (0.1/1)	I (iii)
*STAT3*	**rs4796791** [11]	0.39	0.34	0.022	0.023	0.022	1.23	1.03	1.46	rs4796791 [11]	E (i)
*STAT3*	rs3736164	0.29	0.25	0.033	0.035	0.033	1.23	1.02	1.49	rs1026916 [3] (0.68/1)	E (iii)
*STAT3*	rs17884075	0.38	0.34	0.031	0.028	0.028	1.22	1.02	1.45	rs1026916 [3] (1/1)	E (iii)
*TNFSF14*	rs141395077	0.013	0.027	0.031	0.031	0.030	0.50	0.26	0.94	rs1077667 [3] (0.053/1)	E (iv)
*TNFSF14*	rs117732253	0.004	0.016	0.017	0.019	0.013	0.30	0.11	0.82	rs1077667 [3] (0/0)	I (iii)
*CD40*	rs1883832	0.30	0.26	0.043	0.046	0.042	1.22	1.01	1.47	rs4810485 [11] (1/1)	E (iii)
*CD40*	**rs4810485** [11]	0.30	0.26	0.048	Na	0.047	1.21	1.00	1.46	rs4810485 [11]	E (i)
*CD40*	rs3746821	0.14	0.11	0.049	0.047	0.047	1.30	1.00	1.68	rs6032662 [3] (0.3/0.9)	I (v)
*CD40*	rs41282788	0.013	0.03	0.005	0.008	0.005	0.42	0.22	0.79	rs6032662 [3] (0.09/0.8)	E (iv)
*CYP24A1*	rs6068816	0.14	0.10	0.003	0.005	0.003	1.48	1.14	1.93	rs2585447 [3] (0.04/0.8)	I (iii)
*CYP24A1*	rs2259735	0.38	0.42	0.047	0.040	0.046	0.84	0.70	1.00	rs2585447 [3] (0.08/0.48)	E(ii)
*CYP24A1*	**rs2248137** [3]	0.36	0.40	0.045	0.036	0.041	0.83	0.70	0.99	rs2248137 [3]	E (i)
*CYP24A1*	rs2762943	0.10	0.07	0.009	0.009	0.008	1.53	1.11	2.09	rs2585447 [3] (0.039/0.10)	E (iii)

* MAF: minor allele frequency in the DNA resequencing cohort. + Details of linkage disequilibrium (LD) for CXCR5 variants are provided in Supplementary Figure S2. Genome-wide association studies (GWAS) MS single nucleotide polymorphisms (SNP) LD (*r*^2^/D’) refers to the LD between the previously MS associated variant (reference in brackets) and the reported SNPs in this work. The LD has been calculated using EUR population of 1000 Genome project. Criteria of exclusion (E) or inclusion (I) to the validation phase are described in the Materials and Methods section. In bold are the already known MS associated variants. References for the SNPs associations with MS are shown in brackets.

**Table 2:** Description and functional consequences of MS-associated variants selected for genotyping in an independent cohort.

Gene	Official Full Name	SNP (Ref./Alt.)	Location (hg19)	Origin	Location
*PHLDB1*	pleckstrin homology like domain family B member 1	rs34026809 ** (G/C)	chr11:118480695	IMSGC 2019	Intronic variant
		rs6589706 ** (A/G)	chr11:118747813	IMSGC 2019	Regulatory region variant
*CXCR5*	C-X-C motif chemokine receptor 5	rs10892307 * (C/G)	chr11:118754649	DNA Resequencing	5 prime UTR variant
*CXCR5*	C-X-C motif chemokine receptor 5	rs3922 * (A/G)	chr11:118765600	DNA Resequencing	3 prime UTR variant
*BCL9L*	B cell CLL/lymphoma 9 like	rs149114341 ** (G/A)	chr11:118783424	IMSGC 2019	Intronic variant
*LTBR*	lymphotoxin beta receptor	rs3759323 * (C/T)	chr12:6485436	DNA Resequencing	Intronic variant
*EEF1AKMT3*	EEF1A lysine methyltransferase 3	rs701006 ** (A/G)	chr12:58106836	IMSGC 2019	Intronic variant
*TSFM*	Ts translation elongation factor, mitochondrial	rs1599932 * (G/A)	chr12:58177943	DNA Resequencing	Intronic variant
		rs12588969 ** (C/G)	chr14:103230758	IMSGC 2019	Regulatory region variant
*TRAF3*	TNF receptor associated factor 3	rs74082969 * (A/G)	chr14:103375540	DNA Resequencing	3 prime UTR variant
		rs1026916 ** (A/G)	chr17:40529835	IMSGC 2019	Intronic variant
*STAT3*	signal transducer and activator of transcription 3	rs141732716 * (G/A)	chr17:40469832	DNA Resequencing	Intronic variant
*TNFSF14*	TNF superfamily member 14	rs117732253 * (G/C/T)	chr19:6661631	DNA Resequencing	Downstream variant
*CD40*	TNF receptor superfamily member 5	rs6065926 ** (A/G)	chr20:44735854	IMSGC 2019	Intergenic variant
*CD40*	TNF receptor superfamily member 5	rs3746821 * (G/A)	chr20: 44755111	IMSGC 2019	Intron variant
		rs2585447 ** (T/C)	chr20:52744437	IMSGC 2017	Intergenic variant
*CYP24A1*	cytochrome P450 family 24 subfamily A member 1	rs6068816 * (C/T)	chr20:52781091	DNA Resequencing	Synonymous variant
		rs2762943 * (G/T)	chr20:52790786	DNA Resequencing	Upstream variant

All variants included in the genotyping phase of the study are presented. * Polymorphisms selected from the DNA resequencing cohort. ** Variants from the last IMSGC meta-analysis [3]. rs6065926 is proxy of rs6032662 (variant from the IMSGC meta-analysis [3]). The remaining described polymorphisms constitute those associated with MS in the IMSGC large-scale analysis performed in 2019 and included in our study for conditional regression analyses [3].

**Table 3:** Statistical analysis of genotyping phase data to test for variants-MS association.

Gene	Chr	SNP	Minor Allele	Fisher’s Exact Test (*p*-Value)	OR (95% CI)
***CXCR5***	11	rs34026809 **	C	0.6674	0.9568 (0.78–1.17)
	11	rs6589706 **	A	**0.001902**	1.14 (1.05–1.24)
	11	rs10892307 *	G	**1.37 × 10^−6^**	0.77 (0.69–0.86)
	11	rs3922 *	G	0.5468	0.97 (0.9–1.06)
	11	rs149114341 **	A	**0.0001419**	0.64 (0.51–0.8)
***LTBR***	12	rs3759323 *	T	0.7924	0.98 (0.88–1.1)
***TSFM***	12	rs701006 **	A	**0.009082**	0.89 (0.81–0.97)
	12	rs1599932 *	A	**0.001267**	0.85 (0.77–0.94)
***TRAF3***	14	rs12588969 **	G	**0.0002832**	1.18 (1.08–13)
	14	rs74082969 *	G	0.713	1.05 (0.82–1.33)
***STAT3***	17	rs1026916 **	A	0.07222	1.08 (0.99–1.18)
	17	rs141732716 *	A	0.2896	1.19 (0.86–1.66)
***TNFSF14***	19	rs117732253 *	C	0.2606	0.8 (0.54–1.18)
**CD40**	20	rs6065926 **	A	**0.0002712**	1.19 (1.08–1.3)
	20	rs3746821 *	T	**0.03327**	1.15 (1.01–1.3)
***CYP24A1***	20	rs2585447 **	C	0.7756	1.02 (0.91–1.14)
	20	rs6068816 *	T	0.2994	0.94 (0.82–1.06)
	20	rs2762943 *	T	**0.04738**	1.16 (1–1.35)

SNPs differentially distributed between MS patients and HD are shown in bold. OR: odds ratio. CI: confidence interval. * Polymorphisms selected from the DNA resequencing cohort. ** Variants from the last IMSGC meta-analysis [3].

**Table 4:** Conditional logistic regression of variants of three MS associated regions in the validation dataset.

SNP	Reference (Minor) Allele	*p*-Value	OR (SD)	CI (95%)	*p*-Value	OR (SD)	CI (95%)	*p*-Value	OR (SD)	CI (95%)
***CXCR5***										
		**Conditioned ^†^ to rs10892307**			**Conditioned ^†^ to rs6589706**			**Conditioned ^†^ to rs149114341**		
rs34026809 **	C	0.94	0.99 (0.1)	0.81–1.22	0.57	0.94 (0.1)	0.76–1.16	0.67	0.98 (0.05)	0.86–1.08
rs6589706 **	A	0.26	1.05 (0.05)	0.96–1.16	-	-	-	**0.008**	1.12 (0.04)	1.03–1.22
rs10892307 *	G	-	-	-	**0.0001**	0.79 (0.06)	0.89–0.70	**7.84 × 10^−5^**	0.80 (0.05)	0.72–0.89
rs3922 *	G	0.53	1.03 (0.04)	0.94–1.12	0.51	1.03 (0.04)	0.94–1.13	0.88	1 (0.04)	0.92–1.1
rs149114341 **	A	**0.009**	0.73 (0.12)	0.58–0.93	**0.0008**	0.68 (0.05)	0.85–0.54	-	-	-
***TSFM***										
		**Conditioned ^†^ to rs701006**			**Conditioned ^†^ to rs1599932**					
rs701006 **	A				0.6882	0.97 (0.07)	0.85–1.11			
rs1599932 *	A	0.065	0.87 (0.08)	0.88–1.11						
**CD40**										
		**Conditioned ^†^ to rs6065926**			**Conditioned ^†^ to rs3746821**					
rs6065926 **	A				**0.0032**	1.18 (0.06)	1.01–1.32			
rs3746821 *	T	0.91	1.01 (0.08)	0.87–1.17						

* Variants extracted from the resequencing cohort. ** Variants from the last IMSGC meta-analysis [3]. ^†^ Conditional logistic regression adjusted to an additive model to analyze the dependence of the different variants to contribute to the risk of developing MS. OR: Odds Ratio. SD: standard deviation. CI: 95% confidence interval.

## Supplementary Materials

The following are available online at https://www.mdpi.com/2077-0383/9/3/625/s1, Figure S1: Gating strategy for flow cytometry analysis in PBMC from MS patients. Figure S2: Linkage disequilibrium between the previous reported associated variants at the *CXCR5* locus and the newly found in the present work. Table S1: Demographic and clinical characteristics of the MS patients and healthy controls included in the resequencing cohort. Table S2: Demographic and clinical characteristics of the MS patients and healthy controls included in the validation cohort. Table S3: PCR primers sequences designed for the specific amplification of genomic DNA fragments containing the four SNPs of interest as a first step for the dual luciferase reporter assay. Table S4: Demographic and clinical characteristics of MS patients included in the PCR study to measure mRNA expression levels for CXCR5 and classified according to the presence or absence of the minor allele for rs10892307. Table S5: Demographic and clinical characteristics of MS patients included in the flow cytometry study to determine the expression of CXCR5 in different PBMC populations and classified according to the presence or absence of the minor allele for rs10892307.
