# Supplementary material for: A New Risk Variant for Multiple Sclerosis at 11q23.3 Locus Is Associated with Expansion of CXCR5+ Circulating Regulatory T Cells
Source: J Clin Med. 2020 Feb 26;9(3):625. doi: 10.3390/jcm9030625 (PMC7141122; doi:10.3390/jcm9030625)
Supplement: Supplementary file 1 [file jcm-09-00625-s001.pdf]

Supplementary Content

Supporting Methods.

The gene expression profiling was determined in peripheral blood mononuclear cells (PBMC) from 17 untreated relapsing-remitting MS (RRMS) patients [mean age (standard deviation): 32.9 years (7.3); % females: 70.6%; disease duration: 7.5 years (4.5)] and 15 healthy controls [32 years (6.0); 66.7% females) using microarrays (*Affymetrix Human Exon 1.0 ST expression arrays*). Total RNA was extracted from PBMC and gene expression microarrays were processed following the manufacturer's instructions. Preprocessing was performed with an integrated three-step approach: background correction, normalization (iterPLER), and summarization (Sketch-Quantile) implemented in the Affymetrix Expression Console. The selection of differentially expressed genes between MS patients and healthy control was based on a linear model analysis with empirical Bayes moderation of the variance estimates. P-values were adjusted to obtain control over the false discovery rate (FDR). Only differentially expressed genes between MS patients and healthy controls with adjusted p-values ≤0.05 were considered for further studies.

The following genes associated with MS risk were found to be differentially expressed between RRMS patients and controls and hence included as part of criterion 1 for resequencing in order to identify rare variants:

- *RGS1*: regulator of G protein signaling 1.
Up-regulated in MS patients versus controls.

- *TIMMDC1*: translocase of inner mitochondrial membrane domain containing 1.
Down-regulated in MS patients versus controls.

- *HHEX*: hematopoietically expressed homeobox.
Down-regulated in MS patients versus controls.

- TRAF3: TNF receptor associated factor 3.
Up-regulated in MS patients versus controls.

- TNFSF14: TNF superfamily member 14.
Down-regulated in MS patients versus controls.

Supplementary Table S1. Demographic and clinical characteristics of the MS patients and healthy controls included in the resequencing cohort.

**Characteristics**	**MS patients**	**Healthy controls**
n	524	546
Female/male (%female)	310/214 (59.2%)	316/230 (57.9%)
Age, years[1]	39.5 (10.5)	40.1 (12.7)
Age at onset[1,2]	30.1 (9.7)	-
MSSS[1,3]	3.6 (3.0)	-

[1]Data are expressed as mean (standard deviation). [2]Data are available from 495 MS patients. [3]Data are available from 474 MS patients. All patients included in the study had relapse-onset MS (patients with relapsing-remitting MS and secondary progressive MS).

Supplementary Table S2. Demographic and clinical characteristics of the MS patients and healthy controls included in the validation cohort.

**Characteristics**	**MS patients**	**Healthy controls**
N	3450	1688
Female/male (%)	69/31	62/38
Age (years*)	39.9 (12.8)	42.6 (13.0)
Clinical form (RO/PP) (%)	91.6/8.4	-

*Data are expressed as mean (standard deviation). RO: relapse-onset MS (includes patients with relapsing-remitting MS and secondary progressive MS). PP: primary progressive MS.

Supplementary Table S3. PCR primer sequences designed for the specific amplification of genomic DNA fragments containing the four SNPs of interest as a first step for the dual luciferase reporter assay.

**Polymorphisms and coordinates hg19**	***Forward primer***	***Reverse primer***	**Amplicon**
**rs10892307**<br>chr11:118754513-118754812	TCCACAGTGGGAGAGGATTC	CCTACTGCCTCAGGAGACGA	300 pb
**rs11602393**<br>chr11:118750804-118751192	CTGGAGGCTTGGAGAGAGTG	ATGCCCGGGTAGTTCTGTC	389 pb
**rs3176905**<br>chr11:118755422-118755763	AGCAGGAGGGAGTCAGACAA	TTGGGTGGGCTAAGAAAATG	342 pb
**rs55756957**<br>chr11:118747440-118747671	CACAGCTCCCCTCTCGTTAG	GCTCCTCCATCAGAATCTCG	232 pb

The primers were designed using the Primer3 software (version 0.4.0) and genomic DNA from an individual heterozygous for the four SNPs was amplified.

Supplementary Table S4. Demographic and clinical characteristics of MS patients included in the PCR study to measure mRNA expression levels for *CXCR5* and classified according to the presence or absence of the minor allele for rs10892307.

Characteristics	Presence (CG)	Absence (GG)
N	15	18
Female/male (% women)	9/6 (60.0%)	10/8 (55.6%)
Age (years)[a]	43.4 (14.2)	43.6 (11.0)
Duration of disease (years)[a]	15.6 (9.8)	13.3 (7.0)
EDSS[c]	6.5 (4.0 - 8.5)	6.0 (3.6 - 6.5)
Clinical form (RO/PP) (%)	72.2/27.8	80.0/20.0

[a]Data are expressed as mean (standard deviation). [c]Data are expressed as median (interquartile range). EDSS: Expanded Disability Status Scale. RO: relapse-onset MS (includes patients with relapsing-remitting MS and secondary progressive MS). PP: primary progressive MS.

Supplementary Table S5. Demographic and clinical characteristics of MS patients included in the flow cytometry study to determine the expression of *CXCR5* in different PBMC populations and classified according to the presence or absence of the minor allele for rs10892307.

Characteristics	Presence (CG)	Absence (GG)
N	10	10
Female/male (% women)	7/3 (70.0)	9/1 (90.0)
Age (years)[a]	34.6 (8.4)	39.0 (8.7)
Duration of disease (years)[a]	9.3 (5.2)	12.9 (9.0)
EDSS[c]	1.5 (2.3 - 4.6)	1.4 (2.8 - 5.3)

[a]Data are expressed as mean (standard deviation). [c]Data are expressed as median (interquartile range). EDSS: Expanded Disability Status Scale. All patients included in this cohort were having relapse-onset MS (which includes patients with relapsing-remitting MS and secondary progressive MS).

Supplementary Figure 1.

A

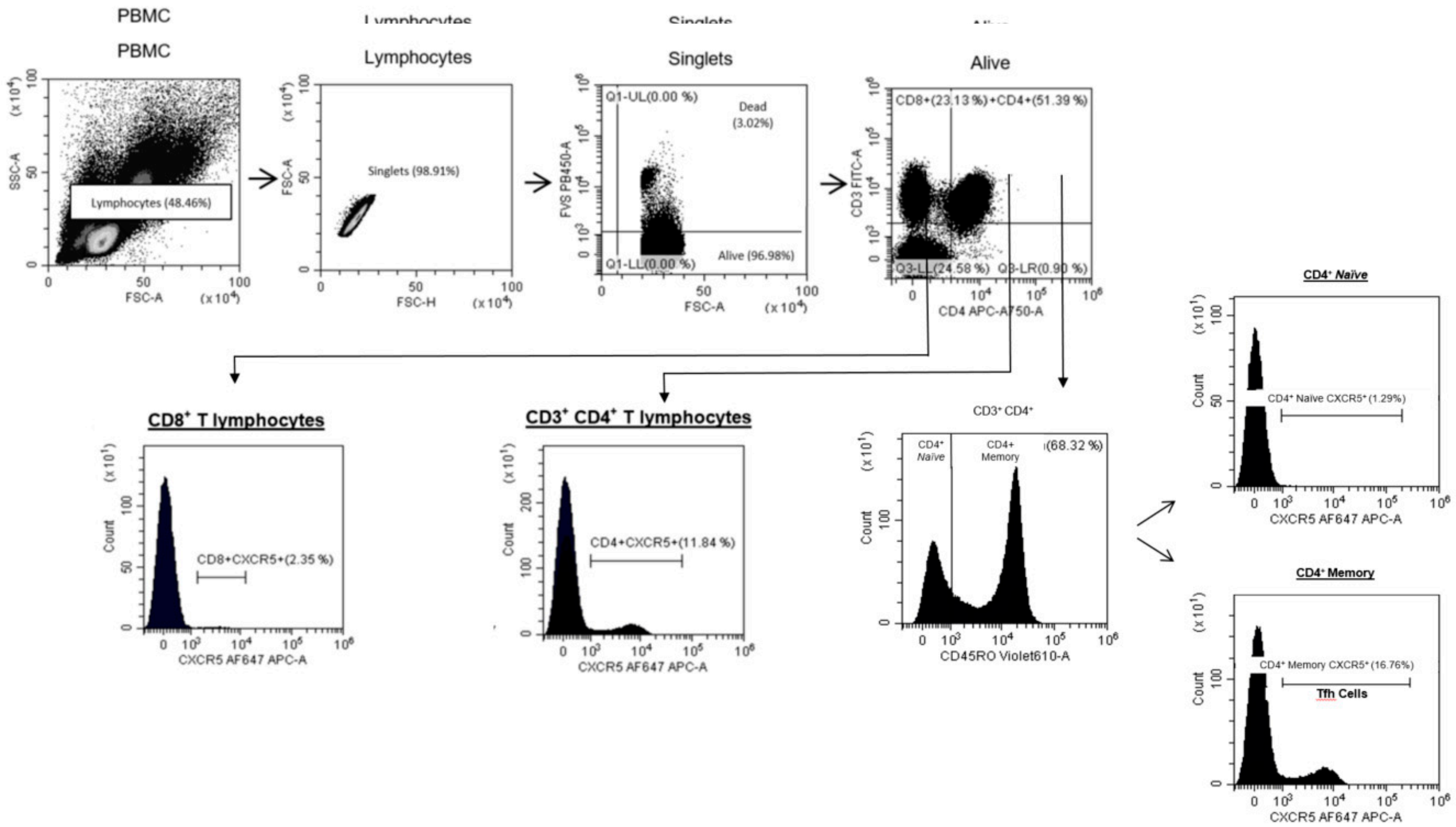

B

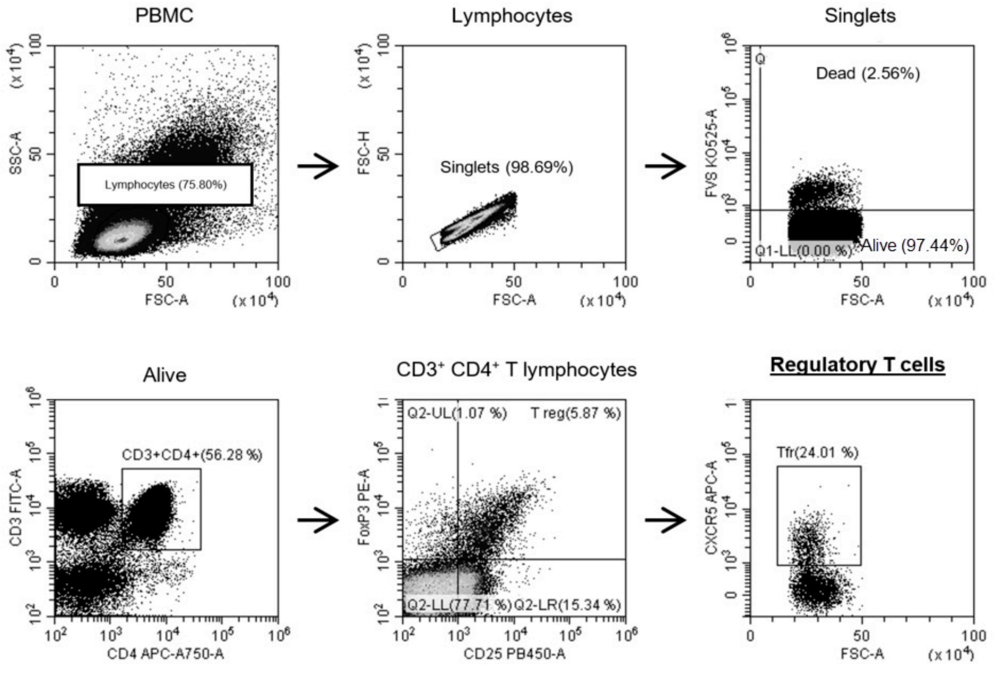

C

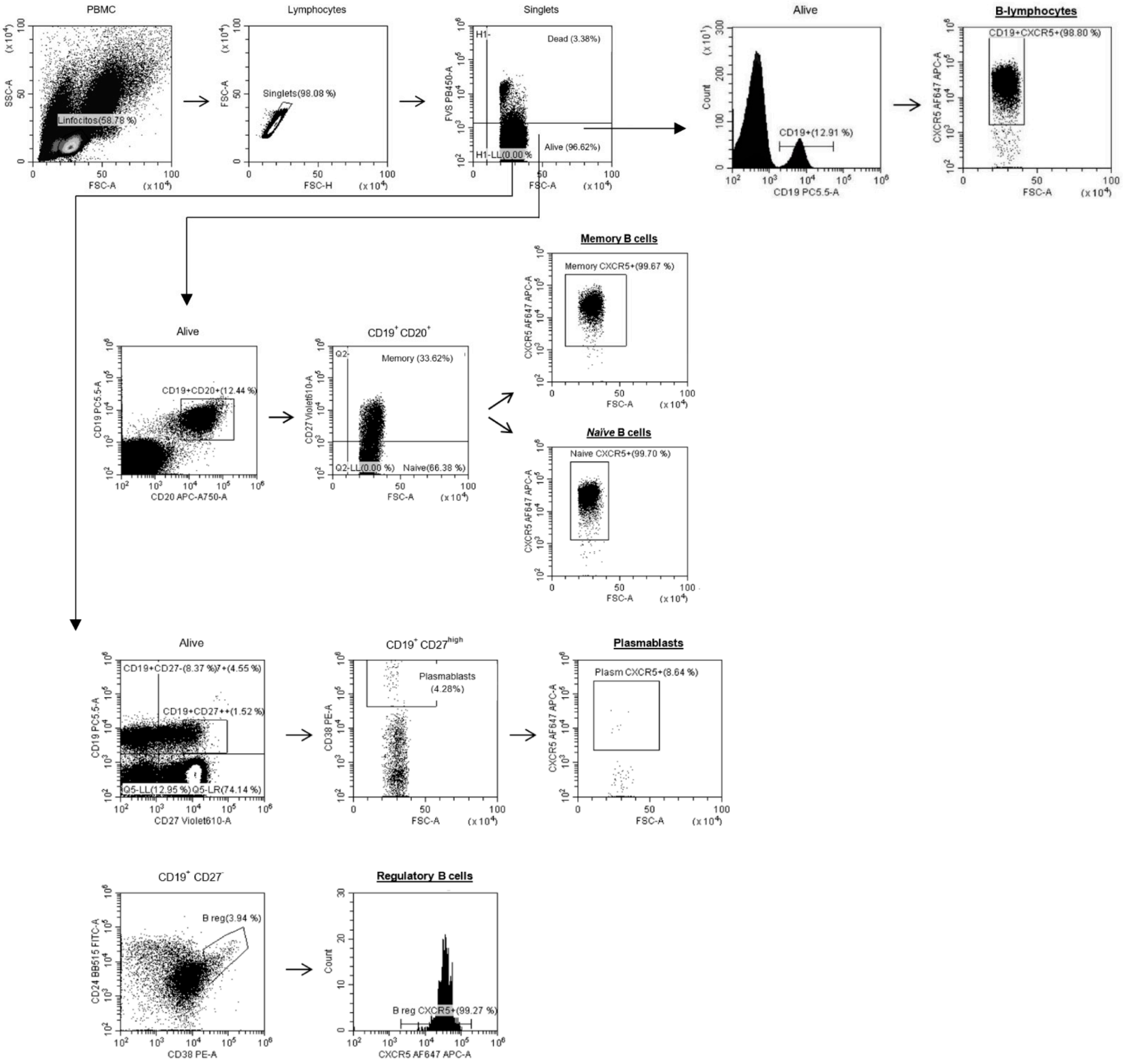

Supplementary Figure S1. *Gating strategy for flow cytometry analysis in PBMC from MS patients. First, in all cases, lymphocytes were ga*ted based on forward and side scatter properties and singlets were selected based on FSC-A vs. FSC-H representation. Following dead cell exclusion by means of a viability dye, three panels were designed with three different gating strategies based on cell surface markers. (A) CD8+ T lymphocytes were defined as CD3+CD4- and CD4+ T lymphocytes as CD3+CD4+. Within the bulk CD4+ T lymphocytes gate, two subsets were defined: naïve CD4+ T cells (CD3+CD4+CD45RO-) and memory CD4+ T cells (CD3+CD4+CD45RO+). CXCR5 expression levels were measured in all described T cells subpopulations. (B) CD3+CD4+ T lymphocytes were gated and, from this subset of cells, regulatory T cells were defined as CD25+FoxP3+. CXCR5 expression was analyzed in regulatory T cells. (C) B lymphocytes were defined as CD19+ cells. Memory B cells were defined as CD19+CD20+CD27+ cells and naïve B cells as CD19+CD20+CD27-. Plasmablasts were gated as

CD19+CD27highCD38high. Lastly, regulatory B cells were gated as CD19+CD27-CD38+CD24+. CXCR5 expression was determined in all described B cells subpopulations.

Supplementary Figure 2.

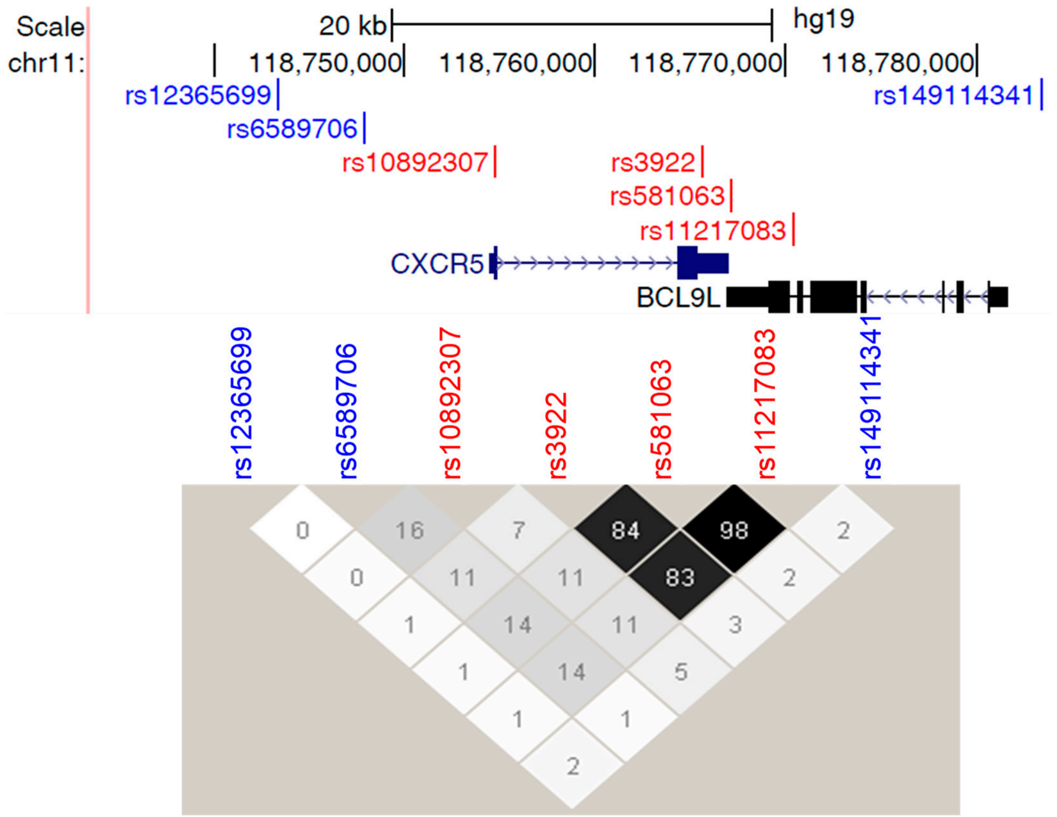

Supplementary Figure S2 Linkage disequilibrium between the previous reported associated variants at the *CXCR5* locus and the newly found in the present work. The picture shows location of the genes and MS risk variants at the locus. Blue color indicates the SNPs reported by the IMSGC study [3]. Red color indicates the SNPs from the present work. At the bottom, the LD (r2) plot of the MS associated SNPs using the European panel of 1,000 Genome Project is represented.
